# Setting the agenda for diabetes research in the state of Qatar

**DOI:** 10.1002/puh2.117

**Published:** 2023-11-15

**Authors:** Amit Mishra, Suresh Babu Kokku, Ioanna Skaroni, Kholoud Ateeq Al Motawaa, Mohammed Al‐Thani, Nicholas Wareham, Abdul Badi Abou‐Samra, Shahrad Taheri

**Affiliations:** ^1^ National Diabetes Strategy Department of Non‐Communicable Diseases Prevention Programs Ministry of Public Health Doha Qatar; ^2^ Qatar Metabolic Institute Hamad Medical Corporation Doha Qatar; ^3^ Department of Non‐Communicable Diseases Prevention Programs Ministry of Public Health Doha Qatar; ^4^ Public Health Department Ministry of Public Health Doha Qatar; ^5^ MRC Epidemiology Unit University of Cambridge Cambridge UK; ^6^ Weill Cornell Medicine—Qatar Doha Qatar; ^7^ Weill Cornell Medicine New York New York USA

**Keywords:** diabetes mellitus, non‐communicable diseases, Qatar, Middle East and North Africa, research agenda

## Abstract

**Background:**

The burden of non‐communicable diseases, including diabetes, is high in the Middle East and North Africa (MENA) region. Qatar (a MENA country) has a high prevalence of diabetes (16.7%). Over the past 20 years, Qatar has made significant investment to establish a biomedical research infrastructure. This article documents the processes adopted for the development of a national diabetes research agenda for Qatar.

**Methods:**

To develop the diabetes research agenda, a three‐step process was adopted. First, a bibliometric analysis of diabetes‐related research publications was conducted to understand current research and funding patterns. Second, through in‐depth interviews and a national consultative workshop, the challenges associated with diabetes research and their potential solutions were documented. Third, an expert team assimilated the recommendations to finalise the diabetes research agenda for the State of Qatar.

**Results:**

A steadily increasing number of diabetes research publications and collaboration with researchers from 48 different countries was noted. Aetiological research (49%), mainly from cohort studies, dominated research publications. The national diabetes research agenda prioritised five research areas focused on diabetes prevention, early detection, reversal, treatment development and evaluation and system research for improved outcomes. Under each area, a set of research questions were identified to guide the research community to align their research interests with high‐priority research in diabetes.

**Conclusion:**

The national research agenda development process has uncovered some important knowledge gaps and outlined the most impactful areas for diabetes research. Achievement of the objectives of the research agenda requires enhanced collaboration among the research community, sustained research funding and enabling a robust regulatory framework.

## Graphical abstract

Qatar has a high prevalence of diabetes and associated risk factors, such as obesity, unhealthy diet and physical inactivity. To promote high‐impact research into the diabetes and associated risk factors, Qatar has published a national research agenda in 2020. This research article documents the participatory research agenda development process.

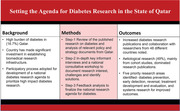

## INTRODUCTION

The prevalence of diabetes mellitus (DM) and prediabetes has increased significantly in adults worldwide over recent decades [1, 2]. In 2017, around 451 million people were estimated to be living with DM globally, and these numbers were expected to increase to 693 million by 2045 [3]. The rising burden of DM is a major healthcare concern resulting in higher cost of care, reduced quality of life and increased mortality [4]. In 2019, DM was directly attributed to an estimated 1.5 million deaths globally [5].

The prevalence of DM in the Middle East and North Africa (MENA) region is 16.2% [6]. With a prevalence of 16.7%, Qatar is among the countries with a high diabetes burden [7]. Qatar has a 24% prevalence of newly detected DM in pregnancy, and of this, about 89.6% is gestational DM [8]. Diabetes prevalence is increasing in Qatar at an alarming rate at younger ages. Qatar also suffers from a higher prevalence of obesity, physical inactivity, smoking and unhealthy dietary habits [9, 10].

The increasing diabetes prevalence globally is associated with rapid urbanisation and dramatic changes in lifestyle [11]. To address the high burden of diabetes in the MENA region, there is a major need for region‐specific impactful research because of the differences in biology, demography, sociocultural factors and financial context between MENA and western countries. Several countries in the MENA region have, therefore, invested significantly in the establishment of biomedical research centres in their academic institutions with an initial focus on basic science and rather less attention to population and clinical research [12]. Despite this investment, the MENA region has been reported to significantly lag in impactful research compared to western countries [13, 14, 15].

In line with Qatar's National Vision (QNV) 2030, Qatar has been increasing its biomedical research capacity through targeted investment. However, much of the research is driven by individual investigators and is not necessarily aligned with the national research needs. The Qatar National Diabetes Strategy (QNDS) has identified these gaps and recommended a plan to promote DM research through a national diabetes research agenda. This article documents the participatory process adopted for the development of national diabetes research agenda and analyses the key patterns and trends in DM research output by exploring the landscape and scope of the published research projects from Qatar.

## METHODS

The national diabetes steering committee, the apex body for implementation of the QNDS, constituted a research sub‐committee in March 2019 with the objective to identify and prioritise DM research. The sub‐committee brought together clinicians, the research community, patient advocates and policymakers to facilitate the development of national diabetes research agenda. The authors of this article included members of the sub‐committee on DM research and were engaged with the research agenda development process.

### Study design

In the first step of this exploratory study, a comprehensive review of the published DM research from Qatar was conducted to understand the current research areas and trends. Moreover, an analysis of policy and strategy documents and research projects of Qatar's Ministry of Public Health (MoPH), and other major health service providers, and academic and research institutions in Qatar was conducted to identify and collate current research activity, research interest and funding patterns for DM research. In the second step, nine purposive in‐depth key informant interviews were conducted with nominated researchers, administrators, regulators, healthcare providers and members of the funding agency to document the research interest and understand associated challenges. This was followed up by a national consultative workshop to collect feedback from diverse stakeholders and to identify potential solutions to research challenges. In the third step, the feedback was reviewed and analysed by two experts from the research sub‐committee to finalise the national research agenda for DM.

### Data sources

For the comprehensive review of research activity, a PubMed search was conducted along with the review of the websites of MoPH, Hamad Medical Corporation (HMC), Primary Health Care Corporation (PHCC), SIDRA Medicine, Weill Cornell Medicine Qatar (WCM‐Q), Hamad Bin Khalifa University (HBKU), Qatar Biomedical Research Institute (QBRI), Qatar Biobank (QBB) and Qatar University (QU). Stakeholders’ views on research conduct, policy, funding, collaboration, capacity building and regulation were obtained through interviews and a national consultative workshop.

### Data collection and processing

**FIGURE 1:**
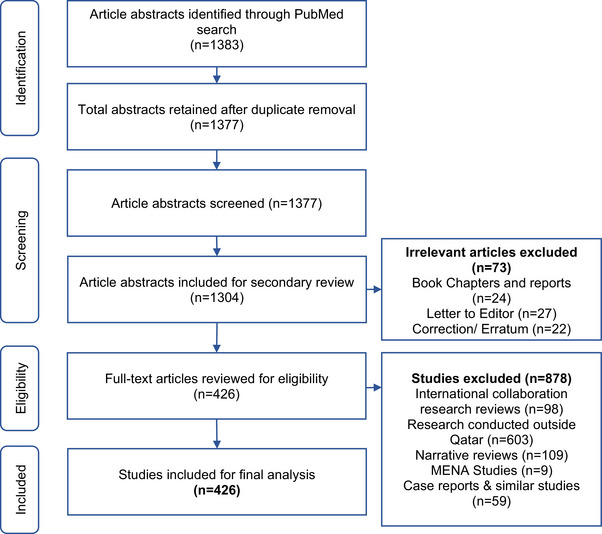
Flow chart of the process of selecting articles reporting diabetes research in Qatar for inclusion in this analysis.

PubMed was searched in January 2021 using following search strategy: ‘(Diabetes) AND Qatar’ [MeSH Terms] to retrieve all publications written in English that included information on DM research in Qatar from beginning until December 2020. A total of 1383 publications were retrieved and reviewed to collect information about the name and number of authors, affiliated institution and country, year of publication, research areas and sources of funding. All diabetes‐related research publications and systematic reviews from Qatar and the MENA region where Qatari nationals were included as study participants were collated. A total of 426 articles were included in final analysis (Figure 1).

Two researchers conducted nine in‐depth interviews with key stakeholders and documented their responses. During the national consultative workshop, findings from comprehensive review and the interviews were presented to gain feedback and explore possible solutions with a diverse group of 44 stakeholders (Table 1). Participants in this workshop were randomly divided into four groups, and each group was assigned a moderator to facilitate the discussions and summarise the identified priority research areas and record suggestions for strengthening the diabetes research ecosystem in the State of Qatar (Table 2).

**TABLE 1:** Type of stakeholders participated in national consultative workshop for diabetes research.

Stakeholders	Description	Number of participants (%)
Government	Senior officials from Ministry of Public Health and National Diabetes Committee	12 (27)
Healthcare organisations	Endocrinologists, specialist physicians, primary care physicians, dietitians, nurses, diabetes educators from Hamad Medical Corporation (HMC), Primary Healthcare Corporation (PHCC) and Sidra Medicine	12 (27)
Academic institutions	Academic and research staff from Weill Cornell Medicine Qatar (WCM‐Q), Qatar University (QU) and Hamad Bin Khalifa University (HBKU)	11 (25)
Funding agency	Representatives from Qatar National Research Fund (QNRF) and Qatar Foundation (QF)	3 (7)
Non‐government organisations	Representatives from Qatar Diabetes Association (QDA)	3 (7)
Research Institutions	Researchers from Qatar Biobank (QBB), Qatar Biomedical Research Institute (QBRI), and Qatar Computing Research Institute (QCRI)	3 (7)

**TABLE 2:** Key diabetes research priorities and research ecosystem gaps identified by stakeholders.

Domain	Themes	Key findings
Priority research areas identified	Diabetes prevention	–Diet, food supplements and their impact on diabetes –Childhood obesity and its role in diabetes
	Early identification	–Genetic studies to identify at risk population –Community‐based screening for diabetes and risk factors –Use of biomarkers for early identification of diabetes –Screening for risk factors in school age children
	Diabetes treatment	–Behavioural and lifestyle interventions for diabetes prevention and treatment –Clinical trials for assessing the effectiveness of complementary and alternative medicine (CAM) on diabetes –Cell therapy and novel treatment interventions –Precision medicine
	Quality of life and Mental Health	–Studies on quality of life of diabetic patients –Understanding the complications of diabetes –Mental health issues associated with diabetes
	Economic evaluation	–Cost–benefit analysis of early disease detection and interventions
	Policy research	–Impact of legislation and regulation on people friendly infrastructure – roads, walkways, sports facilities –Regulation of salt and refined sugar in food
Gaps in research ecosystem	Gaps in research impact optimisation	–General lack of collaboration amongst researchers for sharing research data, best practices and resources –Difficulty in accessing patient‐level clinical datasets for research –Lack of research orientation of clinicians and limited incentives to conduct research –Lack of engagement of patients and their care providers in research prioritisation
	Gaps in research translation	–High focus on researcher driven laboratory and basic science research –Limited avenues to disseminate research findings –Lack of incentives for translation of clinical findings into clinical practice –Limited opportunities to conduct large‐scale clinical trials
	Gaps in funding	–Inadequate funding for diabetes and risk factors research compared to overall diabetes burden in the country –Lack of flexible grants to support junior researchers and proof of concept studies
	Regulatory challenges	–Lack of researcher friendly flexible guidelines and support –Need to regularly review guidelines to accommodate innovations in research

### Data analysis

Bibliometric data was analysed using MS Excel (2019), and for the purpose of this study, all countries affiliated with publication authors were grouped into regions as per the World Bank's list of economies published in June 2020 [16]. The Health Research Classification System (HRCS) published by the UK Clinical Research Collaboration in 2018 was used to classify all selected studies into following eight research areas: underpinning research, aetiology, prevention of disease and conditions and promotion of well‐being, detection, screening and diagnosis, development of treatments and therapeutic interventions, evaluation of treatments and therapeutic interventions, management of diseases and conditions and health and social care service research [17]. Selected studies were further classified based on study type, study population, first author institutional affiliations and funding status.

For responses received during in‐depth interviews of key stakeholders, a thematic analysis was conducted to identify and group related research interest into priority research areas and challenges into research ecosystem gaps. Feedback received from the national consultative workshop was used to further refine the themes. These, along with the recommendations received from stakeholders, were used as input to the national diabetes research agenda development process.

## RESULTS

### Diabetes research trends in Qatar

**FIGURE 2:**
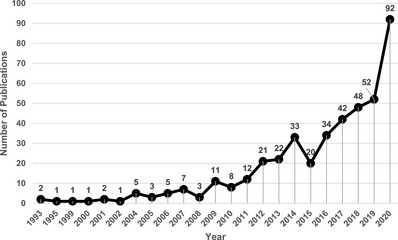
Annual number of articles listed in PubMed reporting diabetes research in Qatar between 1993 and 2020.

The bibliometric analysis indicated that the DM‐related research publications started to emerge from Qatar from 1993 with the total number of articles published each year increasing steadily, particularly from 2016 to 2020 (Figure 2). Cohort studies (43%) were the most common type of publication from Qatar, followed by cross‐sectional (24%) and case–control (9%) studies. Around 8% of studies were systematic reviews (*n* = 21) and randomised control trials (*n* = 11). The remaining articles (16%) were other types of studies, such as case reports, formative research, epidemiological modelling, laboratory and ethnographic studies. Most studies focused on the adult population (75%) with both men and women as study participants. Childhood and adolescent diabetes accounted for 6% of articles, whereas 3% articles studied all age groups; 16% articles did not mention specific age group or gender.

**FIGURE 3:**
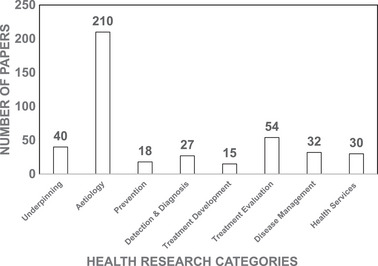
Number of diabetes research articles from Qatar (1993–2020) by health research category defined by the UK Health Research Classification System.

We classified all articles based on the research categories as identified by HRCS. Under HRCS, research studies are classified into eight categories based on the primary objective of the study to assess and compare the allocation of research funds and outputs of different research areas. We used the HRCS to understand overall diabetes research landscape in Qatar (Figure 3) and identify gaps. In our analysis of diabetes research publications, we identified that close to half of the publications from Qatar on diabetes (49%) researched aetiology, followed by the evaluation of treatment (13%), underpinning studies (9%), disease management research (8%) and health services research (7%). Research related to detection and diagnosis (6%), diabetes prevention (4%) and treatment development (4%) were reported in the fewest number of articles.

### Research institutions and collaborations

Qatar has eight international university branch campuses and two national universities (QU and HBKU) running several health research centres and programmes. These institutions and research centres have facilitated the increase in biomedical research publications [18]. For example, from 2012 to 2020, the QBRI published 405 articles [19], WCM‐Q published 1013 articles (2010–2018), SIDRA Medicine published 575 articles (2019–2020) [20], HMC published more than 3000 articles and 38 peer‐reviewed publications were facilitated by data from the QBB [21].

This increase in the number of biomedical research publications was also reflected in DM research, and according to our bibliometric analysis, from 1993 to 2020, a total of 3305 authors participated in the publication of 426 DM‐related articles in 231 journals with an average of 15.2 articles per year. Over time, the number of authors per article has increased with the mean number of authors increasing from seven per article in 1993 to nine in 2020. Fifteen articles published between 2014 and 2020 reported more than 20 authors indicating increased collaboration among researchers. Researchers in Qatar have collaborated with researchers from 48 different countries for about 58% of the publications. These include 19 European and Central Asian countries, 17 Middle East and North African countries, 5 East Asia and Pacific counties, 3 South Asian, 2 North American countries and 1 each from Latin America and the Caribbean and Sub‐Saharan African country (Table S1). However, individually, researchers from the United States (20%) and the United Kingdom (12%) have contributed the highest number of articles. From MENA countries, researchers from the United Arab Emirates contributed to 11% articles followed by researchers from Oman (10%), Egypt (9%) and Bahrain (9%).

### Research funding

The Qatar National Research Fund (QNRF) was the principal funding agency under Qatar Foundation (QF) offering various funding opportunities. Of 785 medical research projects funded by QNRF in 2020, the top recipients were QU (33%), WCM‐Q (25%) and HMC (13%) followed by SIDRA Medicine (6%), HBKU (4%) and others (19%) [22]. Bibliometric analysis also indicated QF as the major source of funding for diabetes‐related publications, which supported 29% of studies. Moreover, about 11% of articles acknowledged study funding support from domestic or external public sector institutions. The private sector contributed funding support to studies that were reported in 6% of articles, and 6% of articles reported joint funding from both the public and private sectors. In about 48% of publications, no research funding was listed. Thirty‐seven publications reported multi‐centre multi‐national cohort studies and phase IV post‐marketing studies funded by eight global pharmaceutical companies.

### Findings from in‐depth interviews and national consultative workshop

Diabetes prevention, early identification, treatment development, quality of life, cost–benefit analysis and policy research were identified as the main research priorities for Qatar. Insufficient funding, limited research impact optimisation and translation into clinical practice and inconsistent research regulatory framework were identified as major gaps in DM research. Stakeholders recommended incentivising researchers to share their data with the research community to enhance collaboration. Also, the development of a diabetes registry, a patient awareness programme focusing on clinical trials, collaboration with international research organisations and sustainable funding were recommended as potential solutions for addressing the current challenges.

### National diabetes research agenda

**FIGURE 4:**
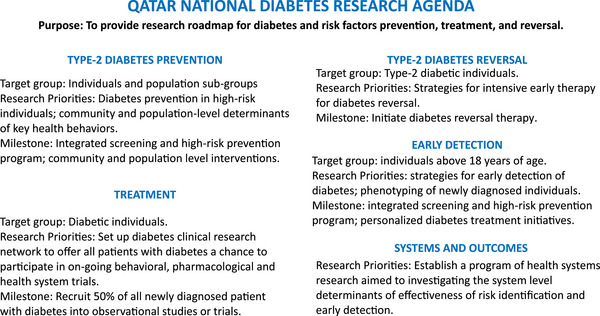
Diabetes research priorities for Qatar as identified by national diabetes research agenda.

The national diabetes research agenda development process uncovered major knowledge gaps and untouched priorities, which can impact the care and prevention of people with DM in the State of Qatar. The agenda was developed to guide scientific investigators and funding agencies alike to focus on five key priority research areas as described below (Figure 4).

Prevention of type‐2 diabetes (T2D): Lifestyle interventions have helped in the prevention of diabetes in multi‐ethnic populations in China, USA, Finland, Japan and India [23, 24, 25, 26, 27]. Recent evidence also suggests that the benefits from lifestyle interventions, such as reductions in diabetes incidence, cardiovascular events, microvascular complications and all‐cause mortality, accrue over longer period of time [28]. There is no singular approach to the prevention of T2D and multiple factors at community and population‐level influence prevention [29]. The research agenda prioritises research on individual and population level prevention strategies that work best in the local context for DM prevention.

Early detection: DM is a slow progressive disease and many studies have indicated that the true biochemical onset of DM may be 3–5 years before the point of clinical recognition [30, 31]. This suggests a window of opportunity in which the disease is detectable by screening and can be managed optimally with benefits outweighing the disbenefits [32]. The research agenda proposes for a research programme into strategies of the early detection of diabetes along with coordinated research for detailed phenotyping of newly diagnosed diabetes individuals as a basis for personalised medicine.

Diabetes reversal: Although it is well known that healthy lifestyle habits are the cornerstone of the prevention of T2D, they are also effective to potentially reverse T2D. This approach is empowering for both patients and clinicians by providing alternative choices to treatment and control of hyperglycaemia. Therefore, the research agenda intends to create a programme of research into strategies for intensive early therapy aimed at DM reversal.

Treatment development: Qatar is making significant investment in early diagnosis and phenotyping of people suffering from prediabetes and diabetes. This will eventually facilitate the sub‐classification of disease and further sequential evaluation of various treatment regimen would help to know what works best for whom. The research agenda proposes to offer all patients with DM a chance to participate in on‐going behavioural, pharmacological and health system clinical trials with an aim to recruit at least half of all with newly diagnosed DM into observational studies or clinical trials.

Systems and outcomes: Prevention and control of DM at population level is affected by the organisation and delivery of the care by health system, such as the use of innovative care models, increased pharmacist involvement in care delivery and education programmes led by healthcare professionals [33]. Moreover, patient‐reported outcomes (PROs) influence in clinical care and research in DM, which are directly reported by patient, without interpretation of the patient's response by a clinician or anyone else [34]. PROs and health systems research would be prioritised to investigate the system level determinants for diabetes risk identification, early detection and care in the State of Qatar.

## DISCUSSION

Bibliometric analysis shows that the research efforts in DM are growing in the State of Qatar with an increase in the number of publications over time, and trends that are consistent with DM‐related research publications globally and from Arab countries [35, 36, 37]. Although there are research output gaps in key areas, such as disease natural history, DM prevention and treatment development and responses, the past research on diabetes prevalence, complications and risk factors in Qatar has facilitated the development of Qatar's National Diabetes Strategy, respective national clinical guidelines for the management of diabetes and an action plan for nutrition and physical activity.

The current research agenda for diabetes aligns with the World Health Organization (WHO) Eastern Mediterranean Region's framework for action on diabetes prevention and control [38] and the national diabetes research priorities of the United Kingdom [39], Canada [40] and Australia [41]. This research agenda also proposes to address emerging research areas, such as omics research and precision medicine. Qatar has tremendous diversity in terms of the ethnicity of the population, and the implementation of research agenda is not without challenges. The most immediate challenge is ensuring the commitment and continuity of funding for diabetes research. Biomedical research usually takes time to translate into clinical outcomes, and sometimes it becomes difficult to justify continued funding. Like any other country with a developing research ecosystem, research in Qatar is investigator‐initiated, and the country has put a greater focus on laboratory research in the past. This needs to evolve, and the country needs to bring forth a mechanism to actively promote and fund most relevant and impactful DM research. This requires institutions to discuss and share their research priorities with other institutions and align their programmes to meet the national needs. This will be a major cultural shift for institutions and researchers as compared to today when they are working in isolation and competing for research funding with each other. A new approach necessitates greater transparency and a platform for research community to collaborate. Acknowledging this challenge, the DM research sub‐committee has organised bi‐weekly DM research webinars to facilitate interaction and knowledge sharing among researchers in Qatar. This, however, is an initial step, and a larger framework needs to be put in place for researcher community to collaborate.

Another major challenge is ensuring the availability of right skill mix for diabetes research. Conquering DM requires an extensive collaboration of expertise from molecular and cell biology to behavioural and social sciences [42]. Bringing in different expertise and reskilling existing researchers would require the implementation of a comprehensive capacity‐building programme. Funders may be persuaded to initiate more innovative funding programmes for the skill building of researchers. Moreover, a dedicated clinician–scientist training programme and national clinical trial unit need to be set up to build research capacities and strengthen research infrastructure. Qatar established a multi‐institutional Academic Health System (AHS) in 2011 to facilitate collaboration among local institutions. Similar collaboration with other international AHS will facilitate in multi‐centre clinical studies and innovations. Most researchers in Qatar are expatriates and creating opportunities for career progression for them will facilitate in the retention of skilled resources in long run.

The Ministry of Public Health in Qatar has recently reformed research regulations, set up a governance mechanism and established a clinical trials registry. Institutional review boards in Qatar have also facilitated in the conduct of scientific research in accordance with international ethical standards. A continuous dialogue with the research community will further help to improve research regulation based on future needs and innovations.

The Qatar Research Development and Innovation (QRDI 2030) was introduced in 2020, providing direction for research and innovation for the next decade in Qatar and intends to address high prevalence diseases through precision medicine and omics research. This is very advanced and complex research, and balancing these with the current research needs would require a calibrated approach. The Gulf Cooperation Council (GCC) countries largely have similar ethnic backgrounds and face similar healthcare challenges, and this provides an opportunity for the research community in these countries to collaborate and align their research to address common challenges associated with the DM in the region.

It is important to acknowledge the limitations of this agenda and approach adopted for its development. This agenda was developed to provide high‐level guidance for research in specific areas and does not specify specific research projects, which need to be further defined. The research agenda does not address how DM research would be funded in future or any mechanism for the rationalisation of funding to support DM research. The agenda was developed by collecting feedback from patient advocacy organisations and not directly from the patients. A greater plan for the engagement of patient community into research needs is planned. Even with these limitations, it is expected that this agenda has great potential to benefit diabetes care and research ecosystem in Qatar.

## CONCLUSION

The State of Qatar has an extensive plan for building strong research and innovation ecosystem in healthcare. Investment in research on diabetes had greatly benefitted many developed countries to reduce diabetes incidence and long‐term complications. To generate evidence in local context, a national diabetes research agenda was prepared with the participation of diverse stakeholders in Qatar. The agenda needs to be adopted by all stakeholders, and funding needs to be ensured for priority diabetes research. Collaboration among different stakeholders, committed funding and strong governance support will facilitate in the achievement of objectives of the research agenda.

## AUTHOR CONTRIBUTIONS

*Conception and design*: Shahrad Taheri, Nicholas Wareham, Abdul Badi Abou‐Samra and Amit Mishra. *Data analysis*: Amit Mishra, Suresh Babu Kokku and Shahrad Taheri. *Drafting the article or revising it critically*: Amit Mishra, Shahrad Taheri, Nicholas Wareham, Abdul Badi Abou‐Samra, Suresh Babu Kokku, Ioanna Skaroni, Kholoud Ateeq Al Motawaa and Mohammed Al‐Thani. All authors approved the final version submitted.

## CONFLICT OF INTEREST STATEMENT

The authors declare no conflicts of interest.

## FUNDING INFORMATION

No funding was received for this work. There was no input from any funding source in the work presented.

## ETHICS STATEMENT AND CONSENT TO PARTICIPATE

The work did not require ethical approval or consent.

## Supporting information

Supporting Information

## Data Availability

Any data used within this work will be available from the corresponding author upon reasonable request.

## References

[1] Ogurtsova K , da Rocha Fernandes JD , Huang Y , et al. IDF Diabetes Atlas: Global estimates for the prevalence of diabetes for 2015 and 2040. Diabetes Res Clin Pract. 2017;128:40‐50. 28437734 10.1016/j.diabres.2017.03.024

[2] Guariguata L , Whiting DR , Hambleton I , Beagley J , Linnenkamp U , Shaw JE . Global estimates of diabetes prevalence for 2013 and projections for 2035. Diabetes Res Clin Pract. 2014;103(2):137‐149. 24630390 10.1016/j.diabres.2013.11.002

[3] Cho NH , Shaw JE , Karuranga S , et al. IDF diabetes Atlas: Global estimates of diabetes prevalence for 2017 and projections for 2045. Diabetes Res Clin Pract. 2018;138:271‐281. 29496507 10.1016/j.diabres.2018.02.023

[4] Baena‐Díez JM , Peñafiel J , Subirana I , et al. Risk of cause‐specific death in individuals with diabetes: a competing risks analysis. Diabetes Care. 2016;39(11):1987‐1995. 27493134 10.2337/dc16-0614

[5] World Health Organization . Diabetes Key Facts. https://www.who.int/news‐room/fact‐sheets/detail/diabetes

[6] International Diabetes Federation . Diabetes in MENA [Internet]. https://www.idf.org/our‐network/regions‐members/middle‐east‐and‐north‐africa/diabetes‐in‐mena.html

[7] Supreme Council of Health, Qatar . Qatar STEPWISE Report, 2012 [Internet]. https://www.psa.gov.qa/en/statistics/Surveys/STEPwise_Report.pdf

[8] Bashir M , E Abdel‐Rahman M , Aboulfotouh M , et al. Prevalence of newly detected diabetes in pregnancy in Qatar, using universal screening. PLoS One. 2018;13(8):e0201247. 30074993 10.1371/journal.pone.0201247 PMC6075760

[9] Khondaker MTI , Khan JY , Refaee MA , Hajj NE , Rahman MS , Alam T . Obesity in Qatar: a case‐control study on the identification of associated risk factors. Diagnostics (Basel). 2020;10(11):E883. 10.3390/diagnostics10110883 PMC7693222 33138081

[10] Al‐Thani M , Al‐Thani A‐A , Al‐Mahdi N , et al. An overview of food patterns and diet quality in Qatar: findings from the National Household Income Expenditure Survey. Cureus. 2017;9(5):e1249. 28630807 10.7759/cureus.1249 PMC5472397

[11] Hu FB . Globalization of diabetes: the role of diet, lifestyle, and genes. Diabetes Care. 2011;34(6):1249‐1257. 21617109 10.2337/dc11-0442 PMC3114340

[12] Bazarbachi AA , Khoury SJ , Sayegh MH . Biomedical research in the Arab region [Internet]. Nature Middle Est. November 20 2014. https://www.natureasia.com/en/nmiddleeast/article/10.1038/nmiddleeast.2014.263

[13] Benamer HTS , Bakoush O . Arab nations lagging behind other Middle Eastern countries in biomedical research: a comparative study. BMC Med Res Method. 2009;9:26. 10.1186/1471-2288-9-26 PMC2674457 19374747

[14] Alenzi FQB , Lotfy M , Nasif W , et al. Biomedical research in the Middle Eastern countries: update and insight using SCImago Journal Rank indicator. J Ayub Med Coll Abbottabad (JAMC). 2010;22:100‐105. 22338430

[15] Al‐Kindi S , Al‐Juhaishi T , Haddad F , Taheri S , Abi Khalil C . Cardiovascular disease research activity in the Middle East: a bibliometric analysis. Ther Adv Cardiovasc Dis. 2015;9:70‐76. 25801472 10.1177/1753944715578585

[16] World Bank . World Bank List of Economies [Internet]. https://data.worldbank.org/country

[17] UKCRC . Health Research Classification System [Internet]. https://www.ukcrc.org/

[18] Idoudi S , Ibrahim MIM , Alali F , Billa N . A Bibliometric analysis of pharmaceutical sciences‐related articles in Qatar from 2013–2020. JPRI. 2021;33:116‐126

[19] Hamad Bin Khalifa University . Publications, Qatar Biomedical Research Institute [Internet]. Hamad Bin Khalifa University. https://www.hbku.edu.qa/en/qbri/publications#/filter/656/

[20] SIDRA Medicine . Research Publications [Internet]. SIDRA Medicine. https://www.sidra.org/research/publications

[21] Qatar BioBank . Publications [Internet]. Qatar Bio Bank [Internet]. https://www.qatarbiobank.org.qa/downloads/annual‐reports

[22] Qatar National Research Fund . QNRF searchable database of awarded projects [Internet]. Qatar National Research Fund. https://www.qnrf.org/en‐us/

[23] Uusitupa M . Good news from the Da Qing Diabetes prevention outcome study‐healthy lifestyles result in long‐term cardiovascular benefits. Ann Transl Med. 2019;7(Suppl 8):S368. 32016086 10.21037/atm.2019.08.123 PMC6976420

[24] The Diabetes Prevention Program Research Group . The Diabetes Prevention Program (DPP): description of lifestyle intervention. Diabetes Care. 2002;25(12):2165‐2171. 12453955 10.2337/diacare.25.12.2165 PMC1282458

[25] Lindström J , Ilanne‐Parikka P , Peltonen M , et al. Sustained reduction in the incidence of type 2 diabetes by lifestyle intervention: follow‐up of the Finnish Diabetes Prevention Study. Lancet. 2006;368(9548):1673‐1679. 17098085 10.1016/S0140-6736(06)69701-8

[26] Kosaka K , Noda M , Kuzuya T . Prevention of type 2 diabetes by lifestyle intervention: a Japanese trial in IGT males. Diabetes Res Clin Pract. 2005;67(2):152‐162. 15649575 10.1016/j.diabres.2004.06.010

[27] Ramachandran A , Snehalatha C , Mary S , et al. The Indian Diabetes Prevention Programme shows that lifestyle modification and metformin prevent type 2 diabetes in Asian Indian subjects with impaired glucose tolerance (IDPP‐1). Diabetologia. 2006;49(2):289‐297. 16391903 10.1007/s00125-005-0097-z

[28] Haw JS , Galaviz KI , Straus AN , et al. Long‐term sustainability of diabetes prevention approaches: a systematic review and meta‐analysis of randomized clinical trials. JAMA Intern Med. 2017;177(12):1808‐1817. 29114778 10.1001/jamainternmed.2017.6040 PMC5820728

[29] Gruss SM , Nhim K , Gregg E , Bell M , Luman E , Albright A . Public health approaches to type 2 diabetes prevention: the US National Diabetes Prevention Program and beyond. Curr Diab Rep. 2019;19(9):78. 31385061 10.1007/s11892-019-1200-z PMC6682852

[30] Wareham NJ , Griffin SJ . Should we screen for type 2 diabetes? Evaluation against National Screening Committee criteria. BMJ. 2001;322(7292):986‐988. 11312236 10.1136/bmj.322.7292.986 PMC1120142

[31] Rahman M , Simmons RK , Hennings SH , Wareham NJ , Griffin SJ . How much does screening bring forward the diagnosis of type 2 diabetes and reduce complications? Twelve year follow‐up of the Ely cohort. Diabetologia. 2012;55(6):1651‐1659. 22237689 10.1007/s00125-011-2441-9

[32] Herman WH , Ye W , Griffin SJ , et al. Early detection and treatment of type 2 diabetes reduce cardiovascular morbidity and mortality: a simulation of the results of the Anglo‐Danish‐Dutch study of intensive treatment in people with screen‐detected diabetes in primary care (addition‐Europe). Diabetes Care. 2015;38(8):1449‐1455. 25986661 10.2337/dc14-2459 PMC4512138

[33] Ong SE , Koh JJK , Toh S‐AES , et al. Assessing the influence of health systems on Type 2 Diabetes Mellitus awareness, treatment, adherence, and control: a systematic review. PLoS One. 2018;13(3):e0195086. 29596495 10.1371/journal.pone.0195086 PMC5875848

[34] Marrero DG , Hilliard ME , Maahs DM , McAuliffe‐Fogarty AH , Hunter CM . Using patient reported outcomes in diabetes research and practice: recommendations from a national workshop. Diabetes Res Clin Pract. 2019;153:23‐29. 31128133 10.1016/j.diabres.2019.05.016

[35] Sweileh WM , Zyoud SH , Al‐Jabi SW , Sawalha AF . Bibliometric analysis of diabetes mellitus research output from Middle Eastern Arab countries during the period (1996–2012). Scientometrics. 2014;101:819‐832.

[36] Li D , Dai F‐M , Xu J‐J , Jiang M‐D . Characterizing hotspots and frontier landscapes of diabetes‐specific distress from 2000 to 2018: a bibliometric study. Biomed Res Int. 2020;2020:1‐13. 10.1155/2020/8691451 PMC6985931 32016121

[37] Beshyah WS , Beshyah SA . Bibliometric analysis of the literature on Ramadan fasting and diabetes in the past three decades (1989‐2018). Diabetes Res Clin Pract. 2019;151:313‐322. 30904744 10.1016/j.diabres.2019.03.023

[38] World Health Organization . Regional Office for the Eastern Mediterranean Framework for Action on Diabetes Prevention and Control in the WHO Eastern Mediterranean Region [Internet]. WHO. https://apps.who.int/iris/bitstream/handle/10665/348184/WHOEMNCD149E‐eng.pdf?sequence=1&isAllowed=y

[39] Diabetes UK . Research Strategy 2020–2025 [Internet]. Diabetes UK. https://www.diabetes.org.uk/resources‐s3/public/2020‐09/Diabetes%20UK%20Research%20Strategy%202020‐2025_0.pdf?_ga=2.83326421.213655820.1599229938‐1291575914.1583944276

[40] Diabetes Canada . Diabetes 360° Recommendations [Internet]. Diabetes Canada. https://www.diabetes.ca/DiabetesCanadaWebsite/media/Advocacy‐and‐Policy/Diabetes‐360‐Recommendations.pdf

[41] Australian Government, Department of Health . Australian National Diabetes Strategy 2021–2030 [Internet]. Australian Government, Department of Health. https://www.health.gov.au/sites/default/files/documents/2021/11/australian‐national‐diabetes‐strategy‐2021‐2030_0.pdf

[42] Fradkin JE , Rodgers GP . Diabetes research: a perspective from the National Institute of Diabetes and Digestive and Kidney Diseases. Diabetes. 2013;62(2):320‐326. 23349536 10.2337/db12-0269 PMC3554357

