# Supplementary material for: Setting the agenda for diabetes research in the state of Qatar
Source: Public Health Chall. 2023 Nov 15;2(4):e117. doi: 10.1002/puh2.117 (PMC12039726; doi:10.1002/puh2.117)
Supplement: Supplementary file 1 — Supporting Information [file PUH2-2-e117-s001.docx]

Supplementary Table 1: Number of diabetes research articles from Qatar between 1993-2020 that include collaborating authors from other countries, grouped by region and country.

| Country | Number of Articles | Country | Number of Articles |
| --- | --- | --- | --- |
| Europe & Central Asia | | Middle East & North Africa | |
| Austria | 1 (0.2%) | Algeria | 3 (0.7%) |
| Belgium | 2 (0.5%) | Bahrain | 37 (8.7%) |
| Croatia | 2 (0.5%) | Egypt | 38 (8.9%) |
| Cyprus | 1 (0.2%) | Iran | 3 (0.7%) |
| Denmark | 1 (0.2%) | Iraq | 2 (0.5%) |
| Finland | 1 (0.2%) | Israel | 1 (0.2%) |
| France | 3 (0.7%) | Jordan | 5 (1.2%) |
| Germany | 16 (3.8%) | Kuwait | 32 (7.5%) |
| Greece | 4 (0.9%) | Lebanon | 5 (1.2%) |
| Italy | 17 (4%) | Morocco | 5 (1.2%) |
| Ireland | 2 (0.5%) | Oman | 41 (9.6%) |
| Netherlands | 11 (2.6%) | S. Arabia | 35 (8.2%) |
| Norway | 1 (0.2%) | Sudan | 2 (0.5%) |
| Poland | 1 (0.2%) | Syria | 1 (0.2%) |
| Slovak Republic | 1 (0.2%) | Tunisia | 4 (0.9%) |
| Spain | 2 (0.5%) | UAE | 46 (10.8%) |
| Sweden | 9 (2.1%) | Yemen | 23 (5.4%) |
| Turkey | 6 (1.4%) | South Asia | |
| UK | 53 (12.4%) | India | 2 (0.5%) |
| East Asia & Pacific | | Pakistan | 4 (0.9%) |
| Australia | 8 (1.9%) | Bangladesh | 1 (0.2%) |
| China | 3 (0.7%) | Sub-Saharan Africa | |
| Korea | 1 (0.2%) | Ethiopia | 2 (0.5%) |
| New Zealand | 6 (1.4%) | Latin America & Caribbean | |
| Malaysia | 2 (0.5%) | Brazil | 2 (0.5%) |
| North America | | | |
| Canada | 22 (5.2%) | USA | 85 (20%) |
